# Supplementary material for: MetaRibo-Seq measures translation in microbiomes
Source: Nat Commun. 2020 Jun 29;11:3268. doi: 10.1038/s41467-020-17081-z (PMC7324362; doi:10.1038/s41467-020-17081-z)
Supplement: Supplementary file 10 — Supplementary Data 7 [file 41467_2020_17081_MOESM10_ESM.zip › File2/Confidence_VeryHigh_Taxonomy/320175_out.krona.html]

Javascript must be enabled to view this page.

members
magnitude
magnitudeUnassigned
count
unassigned
taxon
rank

320175\_out

39

2
39
2
superkingdom

SRS019601\_contig\_number\_contig-100\_11423.91185SRS971275\_contig\_number\_contig-100\_22931.134246


SRS075878\_contig\_number\_contig-100\_23994.23995SRS098881\_contig\_number\_13763SRS105082\_contig\_number\_7117SRS142781\_contig\_number\_contig-100\_12669.55727SRS142890\_contig\_number\_11738SRS143014\_contig\_number\_contig-100\_883.884SRS143218\_contig\_number\_contig-100\_21711.21712SRS143423\_contig\_number\_contig-100\_8371.73715SRS144219\_contig\_number\_contig-100\_1353.1354SRS146996\_contig\_number\_contig-100\_11327.46698SRS147557\_contig\_number\_179
11
37
1224
phylum

class
26
1236

order
91347
26


SRS1055050\_contig\_number\_contig-100\_7317.7318SRS971427\_contig\_number\_contig-100\_36728.193392
543
26
2
family

620
1

SRS024132\_contig\_number\_contig-100\_28362.58107
genus

genus
1
544

1
1344959

SRS015794\_contig\_number\_contig-100\_30676.30676
species group

561
22
1
genus

SRS012902\_contig\_number\_contig-100\_5845.5845

21
562
species

SRS012273\_contig\_number\_28179SRS017191\_contig\_number\_13792SRS017521\_contig\_number\_33167SRS018313\_contig\_number\_contig-100\_10971.44132SRS019068\_contig\_number\_contig-100\_25061.203269SRS019129\_contig\_number\_5053SRS046502\_contig\_number\_contig-100\_2247.2248SRS049402\_contig\_number\_12174SRS064276\_contig\_number\_contig-100\_1693.131248SRS076876\_contig\_number\_contig-100\_1541.18171SRS077127\_contig\_number\_contig-100\_299.76251SRS077646\_contig\_number\_contig-100\_3505.3506SRS098717\_contig\_number\_18542SRS1041031\_contig\_number\_8850SRS1055043\_contig\_number\_contig-100\_28633.65326SRS140492\_contig\_number\_13807SRS144362\_contig\_number\_23166SRS146888\_contig\_number\_15065SRS147022\_contig\_number\_12696SRS147151\_contig\_number\_contig-100\_6269.6270SRS148196\_contig\_number\_18933
